# Development of a Simple IFN-γ Release Whole Blood Assay for the Assessment of *Leishmania infantum* Specific Cellular Immunity in Dogs

**DOI:** 10.3390/ani14233464

**Published:** 2024-11-30

**Authors:** Anna Sára Molnár, Andrea Murillo-Picco, Clara Jiménez-Fortunato, Laia Solano-Gallego

**Affiliations:** 1 Department of Immunology, Faculty of Science, Eötvös Loránd University, 1117 Budapest, Hungary; molnar.anna.sara1997@gmail.com; 2 Departament de Medicina i Cirurgia Animals, Facultat de Veterinària, Universitat Autònoma de Barcelona (UAB), 08193 Bellaterra, Spain; dianaandrea.murillop@gmail.com (A.M.-P.); jimenezfclara@gmail.com (C.J.-F.)

**Keywords:** canine, ELISA, immune response, leishmaniosis, cytokine

## Abstract

Canine leishmaniosis (CanL) is a zoonotic disease caused by *Leishmania infantum*, where increased interferon-gamma (IFN-γ) levels are associated with controlling the infection and mild to moderate disease. Therefore, monitoring IFN-γ concentrations is essential for monitoring the immune responses in CanL. This study compared a faster, cost-effective IFN-γ release whole blood assay in tubes (WBA-T) with a standardized version (WBA-S) in 41 dogs at different states of *L. infantum* infection. WBA-T was performed at 24, 48, and 72 h of incubation with three conditions: blood, blood with *L. infantum*-soluble antigen (LSA), and blood with mitogen ConA. WBA-S was performed in plates, with blood diluted and incubated for five days using the same conditions. Supernatants (WBA-S) or plasma (WBA-T) were harvested for IFN-γ measurement by ELISA. No significant differences were observed in terms of IFN-γ concentration between WBA-T and WBA-S under LSA conditions. However, the 48 h incubation period during WBA-T showed the highest median of IFN-γ concentration compared to other incubation periods and WBA-S. The IFN-γ concentrations under ConA stimulation in WBA-S were significantly higher than in WBA-T at all incubation times studied. In conclusion, WBA-T stimulated with LSA at 48 h incubation time was shown to be the most appropriate for assessing IFN-γ production.

## Simple Summary

Canine leishmaniosis (CanL) is a zoonotic disease caused by a parasite that can be transmitted by a mosquito-like insect called a sandfly. In dogs, the disease can range from mild to very severe, depending on the state of the dog’s immune system. For this reason, it is important to detect the infection early. Whole blood assays (WBAs) are easy tests that allow for the rapid detection of immunity in response to a pathogen. As a result, the WBA is an important diagnostic tool for immune monitoring in CanL. In dogs, however, few tests are available to monitor their immune response to pathogens. This study aims to fill this gap by adapting a human-derived WBA technique for canine use. We compare a novel, faster, and cost-effective WBA in tubes (WBA-T) with a standardized, longer version (WBA-S) in dogs at various stages of *Leishmania infantum* infection. The results obtained showed that WBA-T performed similarly to WBA-S. Therefore, by implementing the WBA-T technique, results can be obtained more quickly and simply, which favors the identification of infected animals at an earlier stage, permitting early control of the infection and thus contributing to minimizing this infectious disease.

## 1. Introduction

Canine leishmaniosis (CanL) is an important zoonotic disease, caused by *Leishmania infantum*, an obligate intracellular protozoan. This infection is prevalent in the Mediterranean Basin regions [1], the Middle East [2], China [3], and South America [4]. Transmission of the disease occurs by the bite of an infected female sandfly [5]. After infection of a mammalian host, the flagellated promastigote is phagocyted by immune cells such as macrophages. Internally, it transforms into a non-flagellated amastigote and uses the immune cell to multiply. Eventually, the lysis of immune cells causes the parasites to infect other cells in surrounding tissues [6]. The infected animals play a major role in spreading the disease in endemic areas where the sand fly exists [7]. It is a major concern because, only in the Mediterranean basin, approximately 2.5 million dogs are infected [8], which are believed to be the main reservoirs [7]. In addition, global warming and human activities have contributed to the geographical spread and epidemiology of *L. infantum* infection in Europe, particularly in Spain and Portugal [9,10].

The clinical manifestation of this infection is very broad. A high proportion of dogs present a chronic subclinical infection, while other dogs manifest clinical illness with different degrees of severity. A wide spectrum of the disease exists, from mild to very severe clinical illness. For this reason, a clinical staging was proposed, and sick dogs are classified from mild (stage I) to very severe disease (stage IV) [11]. Therefore, this clinical variability makes diagnosis challenging and complicated, and early detection of infection or disease is extremely difficult [8]. However, the different outcomes of the infection mostly depend on the host’s immune response [12]. Several studies have described healthy infected dogs showing a stronger cellular immune response with higher expression levels of interferon (IFN)-γ and a weaker humoral immune response as opposed to sick dogs [13,14,15,16].

As the immune response has an important role in the outcome of the disease, tracking the *Leishmania*-specific cell-mediated immunity of the host is a determinant in predicting disease outcomes [17]. Different techniques are used to identify the infection of *Leishmania*: by measuring cytokines, for instance, or peripheral blood mononuclear cells (PBMC) assay or whole blood assay (WBA). On the other hand, there are few and poorly standardized assays to evaluate T-cell-mediated immunity responses in dogs [8].

WBA is often used as a diagnostic tool for a variety of human infectious diseases where T-cell-mediated immunity plays an important role, including *Leishmania* spp. infection [18]. It is the most suitable method for measuring cytokines, since they contain all the cell populations and soluble factors required for T-cell activation, and therefore it shows the best mimicry of ex vivo conditions when compared to PBMC, where the samples normally are not devoid of various growth factors and other immune components [19,20]. Moreover, PBMC requires more blood volume and is time-consuming when compared with WBA [21]. Therefore, WBA with soluble *Leishmania* antigen (LSA) followed by cytokine determination is the most suitable technique to provide the detection of human patients with asymptomatic *Leishmania* infection, and so provides a better prediction of the infectious outcome [16].

Even though WBA is shown to be an important diagnostic tool for immune monitoring of human leishmaniasis [19], it is not widely used in veterinary medicine, specifically in dogs [22]. Previous research has described the use of this technique in dogs in a vaccine trial against leishmaniosis [23]. WBA has been shown to be a useful tool to follow up on the immunogenicity of the dogs before and after vaccination [23]. Likewise, some researchers have used the technique to identify cytokine patterns, such as IFN-γ and IL-10, in dogs with clinical leishmaniosis [15]. It helped to have more immunological data of sick dogs with different stages of disease severity due to *L. infantum* natural infection at diagnosis and during treatment [15,24] as well in healthy infected dogs [15].

On the other hand, WBA in CanL has a long incubation period and requires significant effort and investment, since the heparinized blood is diluted to a ratio of 1:10 with medium on a culture plate and incubated for at least 5 days, based on the methodology described so far [15,23,24]. Moreover, it also needs various supplies, which makes the technique costly to use. Due to these facts, the test could be challenging to implement and faces limitations of its frequency of use in the clinical setting. These may be some of the reasons why this technique has not been widely used in veterinary medicine and is only used for research purposes and not in the veterinary clinical setting.

However, previous studies have already developed and optimized WBA in tubes without medium, using LSA to detect IFN-γ production against visceral leishmaniasis (VL) in humans with 20–24 h incubation times [25]. It is still a well-developed technique to detect asymptomatic infected human patients with leishmaniasis and measure chemokines or cytokines released by activated *Leishmania* antigen-specific T lymphocytes [26]. Even so, this way of performing WBAs is not widely used for the study of *Leishmania* infection in dogs, as just one study used this technique, where WBA was performed in tubes without medium and incubated for 16–20 h [22].

In the present study, we sought to identify a reliable, fast, and feasible ex vivo method to assess canine cellular immune responses to *L. infantum* antigen. Therefore, the aim of this study was to investigate a faster, more practical, and cheaper parasite-specific IFN-γ release WBA in tubes (WBA-T) compared with a standardized and longer WBA (WBA-S) in dogs in different states of *L. infantum* infection.

## 2. Materials and Methods

### 2.1. Dogs and Sampling

The study involved forty-one dogs of different age, sex, and breed from Catalonia, Spain. They were recruited in a prospective study and blood samples were collected between October 2022 and April 2023. Each dog underwent a full physical examination, alongside enzyme-linked immunosorbent assay (ELISA) testing aimed at detecting antibodies against *L. infantum* antigen, as well as measurement of parasite-specific IFN-γ after WBA. The diagnosis of clinical leishmaniosis was established based on clinical signs and/or clinicopathological abnormalities compatible with the disease and a seropositive result by in-house ELISA [8].

The dogs were classified into 4 groups based on their physical examination, clinical history, antibody levels, and IFN-γ production: (1) healthy seronegative/non-IFN-γ producers (*n* = 14), (2) healthy seronegative or seropositive/IFN-γ producers (*n* = 20), (3) sick seropositive/non-IFN-γ producers (*n* = 2) and (4) sick seropositive/IFN-γ producers (*n* = 5).

Twelve mL of blood was drawn from each dog, obtained either from the jugular or external cephalic vein. Of this total volume, 10 mL was carefully collected into sodium heparinized tubes (Vacutainer^®^, Plymouth, UK). Additionally, 2 mL of blood was collected into serum tubes (Eurotubo^®^, Rubí, Spain) for further serological assessments.

### 2.2. Whole Blood Assay (WBA)

Aerobic, sterile, round-bottom 5 mL culture tubes were used to perform the WBA-T (biowest^®^ Kansas, MO, USA). Three different conditions were established during each incubation time: (1) blood with phosphate-buffered saline (PBS) (negative control), (2) blood with soluble *L. infantum* antigen (LSA) at a final concentration of 10 µg/mL, and (3) blood with mitogen concanavalin A (ConA) (100 mg Medicago^®^, Uppsala, Sweden) at a final concentration of 10 µg/mL (positive control). In each tube was added 1 mL of heparinized blood and 10 μL of PBS, LSA, or ConA. The tubes were incubated for 24 h, 48 h, and 72 h in a 37 °C and 5% CO_2_ incubator.

To perform the WBA-S, flat-bottom plates were used. Three hundred μL heparinized blood was diluted to a ratio of 1:10 with a volume of 2.7 mL of medium RPMI 1640 with stable glutamine and 25 mM hepes (biowest^®^, Kansas, USA) supplemented with 100 μg/mL of penicillin, 100 μg/mL streptomycin (Life technologies™, New York, CA, USA) and 10% of fetal bovine serum of premium South American origin (biowest^®^, Kansas, USA). The same three experimental conditions, each with identical final concentrations, were conducted, with the only deviation being the negative control, which consisted of blood with medium alone. Subsequently, the plates were placed in a 37 °C, 5% CO_2_ incubator for 5 days.

Following the incubation period, the samples underwent centrifugation at 562.5× *g* for 10 min (WBA-T) or 300× *g* for 10 min (WBA-S). The resulting supernatant (for WBA-S) or plasma (for WBA-T) was harvested, and all samples were immediately stored at −20 °C until analysis.

For the purpose of this study, the results from LSA were analyzed using data from all 25 IFN-γ-producing dogs. Furthermore, the results from ConA were analyzed for a total of 41 dogs.

### 2.3. IFN-γ Enzyme-Linked Immunosorbent Assay (ELISA)

IFN-γ concentrations were measured from supernatants (WBA-S) or plasma (WBA-T) with sandwich ELISA (DuoSet^®^ ELISA by Development System R&DTM, Abingdon, UK) using 96 well-cell flat-bottom plates (Costar^®^ Corning, NY, USA) following the manufacturer’s instructions with slight modifications. The standard curve started at 4000 pg/mL, with subsequent two-fold dilutions until reaching 31.25 pg/mL. Additionally, four duplicated blanks were placed on the plate. WBA-S and WBA-T samples treated with ConA were diluted 1:1 with the sandwich ELISA kit diluent. When the values of the sample with ConA were outside of the curve, we repeated the sample with a higher dilution. The reaction was stopped at approximately 20 min. To stop the reaction, 50 µL of N2 H_2_SO_4_ was added per well. The optical density was read in an automatic micro-ELISA reader (ELISA Reader HEALES MB-580, Shenzhen, China) at the wavelengths of 450 nm and 560 nm for the correction of optical imperfections in the plate. All supernatant or plasma samples were duplicated on the plate to ensure data consistency. Furthermore, to minimize potential variations between plates, all samples obtained from a single dog were processed on the same plate. The standard curve and quantification of cytokine concentrations were determined using a four-parameter logistic curve generated by the MyAssays program (http://www.myassays.com/). Plates were repeated if the R^2^ value of the standard curve was below 0.98, ensuring the reliability and accuracy of the data. For cytokine analysis, the values used were obtained by subtracting the result of the medium only (negative control WBA-S) or blood with PBS only (negative control WBA-T) from each of the remaining two conditions (LSA and ConA). This subtraction method allowed for the isolation and quantification of cytokine levels specifically induced by stimulation with LSA or ConA, thus providing a more accurate assessment of the immune response of the experimental samples.

All dogs included in the study were confirmed to be IFN-γ-producers following ConA stimulation, as this served as a positive control. Dogs that did not produce IFN-γ after stimulation with ConA were excluded from the study.

Dogs were classified as IFN-γ producers when *L. infantum*-specific IFN-γ concentrations were ≥100 pg/mL in WBA-S after subtracting the value obtained in the negative control [27].

### 2.4. Enzyme-Linked Immunosorbent Assay for the Detection of Antibodies Against L. infantum Antigen

Antibody levels were measured with an *L. infantum* in-house ELISA protocol previously described for dogs [8]. Dogs’ plasma or serum was diluted to 1:800 in phosphate-buffered saline (PBS)-0.05% Tween 20 containing 1% dry milk and incubated in an *L. infantum* antigen-coated plate (20 μg/mL) for an hour at 37 °C in a humid chamber. Then, it was incubated again for an hour at 37 °C with Protein A conjugated to horseradish peroxidase (1:30.000 dilution; Sigma-Aldrich). Ortho-phenylene-diamine (OPD) and stable peroxide substrate buffer were added to the plate (SIGMAFAST OPD; Sigma-Aldrich, Darmstadt, Germany), and the reaction was stopped with 50 μL of 2.5 M H_2_SO_4_. The optical density was measured with an ELISA reader (ELISA Reader HEALES MB-580, Shenzhen, China) at 492 nm wavelengths. All the samples were run in duplicates on each ELISA plate. The results were measured in ELISA units (EU), standardized against the positive dog serum employed as the calibrator, with a reference point set arbitrarily at 100 EU. Serum or plasma was classified as high positive when the EU was equal to or higher than 300 EU; medium positive when it was equal to or higher than 150 EU and less than 300 EU; low positive when it was lower than 150 EU and equal to or higher than 35 EU; doubtful when it was lower than 35 EU and equal to or higher than 25 EU and negative when the EU was lower than 25 [14].

### 2.5. Statistical Analysis

The statistical analyses were conducted by the freely available software Jamovi (Sydney, Australia) [28,29,30,31]. To ensure the applicability of parametric statistical tests, a logarithmic neperian (ln) transformation of the data was performed. Subsequently, comparisons among the different incubation times were conducted using repeated-measures ANOVA. In cases where statistically significant differences were detected, the Tukey test was employed as a post hoc analysis to elucidate the specific incubation times between which the differences were observed. Throughout the analyses, data were presented as the median and interquartile range (IQR), offering a robust summary of the central tendency and variability within the dataset. The associations between different clinical states of infection and between breed and sex were tested using Chi-Square tests. The relationship between clinical states of infection and age was assessed using ANOVA. Statistical significance was determined based on a threshold *p*-values were <0.05, indicative of meaningful differences between experimental conditions or groups.

## 3. Results

All data supporting the main conclusions of the present study are displayed in supplementary material Table S1 (Table S1: signalment, clinical data, and IFN-γ concentrations with WBA-T at 24, 48, and 72 h of incubation and WBA-S at 5 days of incubation after stimulations with LSA and ConA).

### 3.1. Clinical and Immunological Characteristics of Dogs

The median age of the 41 dogs recruited for the study was 4.5 years with a range from 1 to 9 years. The study group included 16 female and 25 male dogs. The breeds included were 24 purebred dogs from more than 13 different breeds and 17 mixed breeds. 33 dogs were spayed, representing 89.2% of the study population, while 4 dogs were unspayed, representing the remaining 10.8%. Unfortunately, data on the spay status of a further 4 dogs was missing.

The serological status of the dogs regarding *L. infantum* was determined in each case. Of the 41 dogs, 14 were seronegative and did not produce IFN-γ, while 20 dogs were healthy and either seronegative or seropositive IFN-γ producers. All sick dogs were seropositive. Only 2 of the sick dogs did not produce IFN-γ, while 5 sick dogs produced IFN-γ. The antibody levels were significantly higher in sick dogs compared to healthy dogs (*p* = 0.001). The highest antibody production was detected in sick dogs that produced IFN-γ, while the highest levels of IFN-γ concentration were detected in healthy seronegative/seropositive dogs, that produced IFN-γ (Table 1). The oldest median age was observed in sick dogs not producing IFN-γ, while the youngest median age was observed in healthy dogs not producing IFN-γ (Table 1). No significant differences were observed between the clinical states of *L. infantum* infection and age (*p* = 0.106), breeds (*p* = 0.962), or sexes (*p* = 0.733). Table 1 provides a comprehensive summary of the signalment and immunological tests performed in each group studied.

In the analysis of IFN-γ producers after stimulation with LSA, it was found that the group of healthy dogs, characterized as seronegative/seropositive IFN-γ producers, showed higher IFN-γ median production after being stimulated with LSA when compared with other groups studied. It was observed that the 48 h incubation period during WBA-T showed the highest median of IFN-γ concentration compared to other incubation periods and WBA-S (as detailed in Table 2). A lower median concentration of IFN-γ production was documented in sick IFN-γ-producing dogs when compared with the healthy infected dog group independently of the incubation period used. Interestingly, low IFN-γ production was observed in sick, non-IFN-γ-producing dogs only during the 48 and 72 h incubation periods of WBA-T. Table 2 and Table 3 provide a comprehensive descriptive statistic, demonstrating the clinical characteristics observed during different incubation times for both LSA and ConA stimulation conditions in each group of dogs.

In all groups studied, it was observed that WBA-S had the highest IFN-γ production under ConA stimulation. However, an exception was noted in sick dogs identified as non-producers of IFN-γ, where IFN-γ concentrations were comparatively lower in WBA-S compared to WBA-T incubation times, as detailed in Table 3.

The analysis revealed that the highest median of IFN-γ concentration (pg/mL) was observed in healthy dogs characterized as seronegative/seropositive IFN-γ producers under ConA stimulation conditions at all the incubation times. Furthermore, the lowest levels of IFN-γ production were identified during the WBA-S incubation period among the sick non-IFN-γ producer dogs. Conversely, within the WBA-T incubation periods, the lowest production was observed among the healthy seronegative non-IFN-γ producer dogs (Table 3).

### 3.2. Comparison Between WBA-S and WBA-T After Stimulation with LSA

Of the 41 dogs included in the study, 25 dogs (60.1%) showed IFN-γ production following WBA-S stimulation with LSA. Notably, one dog out of these 25 IFN-γ producers in WBA-S did not demonstrate IFN-γ production at any incubation time in WBA-T, only in WBA-S. No significant differences were observed among the different incubation times (24 h, 48 h, and 72 h) following stimulation with LSA. Additionally, there were no detectable differences between WBA-T at all incubation times compared to a standardized WBA-S under LSA stimulation conditions (*p* = 0.068). The WBA-T 48 h incubation period exhibited a higher median IFN-γ production compared to other incubation times in both WBA-T (24 h and 72 h) and WBA-S (5 days) (*p* = 0.05) (Table 4). Furthermore, the WBA-T 24 h and WBA-T 72 h incubation periods demonstrated lower IFN-γ production percentages compared to the WBA-T 48 h incubation time. The lowest median was observed for both WBA-T and WBA-S during the 24 h incubation period (Table 4).

### 3.3. Comparison Between WBA-S and WBA-T After Stimulation with ConA

The analysis of IFN-γ production following stimulation with ConA revealed that most of the dogs in the study exhibited a robust response (Table 5). As expected, significantly higher IFN-γ concentrations were observed after blood stimulation with ConA compared to LSA (*p* = 0.0068). Differences were detected between WBA-S and all incubation times in WBA-T under ConA stimulation conditions (*p* = 0.0006). For instance, during a 24 h incubation period, the concentrations of IFN-γ were notably different (*p* = 0.009) between WBA-S and WBA-T, measuring 10,970 pg/mL and 4050 pg/mL, respectively. Similarly, during 48 h of incubation, the disparity persisted, with IFN-γ concentrations measuring 5587 pg/mL in WBA-T and 10,970 pg/mL in WBA-S (*p* = 0.006). This trend continued through the 72 h incubation period, with IFN-γ concentrations at 4924 pg/mL in WBA-T and 10,970 pg/mL in WBA-S (*p* = 0.005) (as detailed in Table 5). Notably, only the WBA-T 24 h period did not exhibit a 100% response rate. In the context of WBA-T incubation, the 48 h period was found to be the period with the highest IFN-γ production, compared to the 24 h and 72 h intervals. In contrast, the 24 h time point showed the lowest IFN-γ production among the time periods studied.

## 4. Discussion

In the present study, we did not find a statistically significant difference between the three incubation times tested with WBA-T compared to WBA-S after stimulation with LSA. It is also noteworthy that the most favorable results were observed after 48 h of incubation in WBA-T when compared with WBA-S. To the best of our knowledge, only one study has followed a similar protocol of WBA in tubes in dogs [22]. In this previous study, canine blood samples were incubated for 16 to 20 h [22] with good results. In other previous studies involving human patients with leishmaniasis and detecting different cytokines, WBA tubes containing LSA were incubated at 37 °C for 24 h [26,32]. Variations in culture incubation time may arise across different laboratory settings. Additionally, the initial lower levels of certain cytokines, such as IFN-γ, may be attributed to the requirement for peripheral blood lymphocytes to undergo differentiation into cells capable of producing such cytokine responses after LSA stimulation. As a result, cytokine production may become more detectable after an extended culture period, such as 48 h, compared to a shorter time like 24 h. The extended incubation period promotes the development and activation of lymphocytes, resulting in increased cytokine production as time progresses [33,34]. However, it is noteworthy that incubation periods exceeding 72 h are not advisable due to the gradual depletion of nutrients, the impairment of culture conditions, and a significant decline in cell viability, as these culture tubes were not enriched with traditional culture medium [35]. This observation agrees with our results showing a reduction in cytokine production after 48 h of incubation, indicating that longer incubation beyond this point may not provide additional benefits. For human patients, most protocols prescribe an incubation time of 24 h. This suggests that the difference in IFN-γ production between 24 and 48 h of incubation in humans is not necessarily significant [36]. In contrast, our findings suggest that in dogs, the 48 h incubation period appears to be the most suitable one. This conclusion is drawn from the observation that IFN-γ production does not reach detectable levels during the initial 24 h period, as evidenced by some of our samples. It is also noteworthy that WBA-S, despite having better culture conditions due to the medium, yielded lower IFN-γ concentrations compared to the 48 h incubation time WBA-T. This indicates that a longer incubation period does not necessarily lead to increased cytokine production, even in the presence of the medium. Furthermore, in agreement with previous studies, it has been documented that the highest synthesis of IFN-γ is typically observed after 48 h of incubation in blood collected in heparinized tubes from human patients [37]. Therefore, longer incubation time is not relevant for better outcomes. By utilizing this optimized protocol, clinicians and researchers can reliably evaluate the immune response of dogs to *Leishmania* infection, facilitating timely diagnosis, monitoring, and treatment of this disease.

Interestingly, a low concentration of IFN-γ after LSA stimulation was observed in the sick, non-IFN-γ producer group after 48 and 72 h of incubation in WBA-T, but not in WBA-S. This finding may suggest that WBA-T has greater sensitivity compared to WBA-S. However, given the small sample size, the low concentration of IFN-γ, and the lack of similar results from other groups, this result could be just a measurement error or false positive result. However, further studies might investigate these findings with a higher number of sick non-IFN-γ producer dogs.

Concavalin A is a plant lectin known for its ability to stimulate the mitogenic activity of T lymphocytes and exhibit diverse bioactivities. Consequently, ConA treatment leads to heightened secretion of inflammatory cytokines, including IFN-γ, in blood samples [38]. Therefore, it serves as an effective positive control in experimental setups, owing to its ability to induce robust immune responses. ConA-induced IFN-γ production consistently exceeded the threshold of ≥100 pg/mL at all clinical states of infection and all incubation periods, which was used as a criterion for classifying dogs as IFN-γ producers in response to *L. infantum* [27]. The robustness and consistency of ConA-induced IFN-γ production observed at all clinical states of infection and incubation periods underscores its reliability as a positive control for WBA protocols, confirming its suitability for evaluating the immune response in dogs with leishmaniosis.

The decreased cytokine levels observed in WBA-T compared to WBA-S following ConA stimulation can be explained by the essential components in the medium used in WBA-S. Unlike WBA-T, which does not contain medium and thus lacks a diverse array of nutrients, including carbohydrates, vitamins, amino acids, minerals, growth factors, and hormones, WBA-S incorporates serum that provides a rich milieu for cellular function and cytokine production. Consequently, the reduced cytokine levels in WBA-T underscore the importance of the medium-derived factors in promoting robust immune responses upon ConA stimulation [39]. However, based on our statistical results when we stimulated the blood with LSA, we did not observe significant benefits associated with the use of cell culture media. This may be because ConA binds to glycoproteins on the surfaces of T-cells, which triggers a process like the activation of T-cell receptors [40]. Continued stimulation of T-cells, especially in conditions where no supplementary nutrients are available, can lead to faster T-cell death. Serine deficiency can often lead to cell death. Serine is a vital amino acid that is essential for immune cell proliferation, differentiation, and the production of specific immune cytokines [41]. Serine phosphorylation is critical for IL-12-induced IFN-γ production [42]. Indeed, depletion of serine may lead to reduced IFN-γ production, which may explain the lower levels observed in WBA-T compared to WBA-S following ConA stimulation. The presence of the RPMI medium in WBA-S may further suggest that it supports immune cell function and cytokine production. However, further research is needed to comprehensively clarify this relationship. This means that, although many cells can produce cytokines in the absence of a cell culture medium, there are cases where cells do not release cytokines due to insufficient nutrient supply.

In addition, it is crucial to recognize that various factors, including age and health status, can influence cytokine production in whole blood assays and thus potentially affect the results observed specifically in WBA-T. These observations highlight the complex interplay of different factors in immune responses and emphasize the need to take these complex factors into account in experimental designs and the interpretation of results [43]. These results highlight the complexity of immune responses under different experimental conditions and demonstrate the need for careful consideration of the different factors influencing cytokine production in WBA. Another limitation of the present study is the small number of sick IFN-γ producer and sick non IFN-γproducer dogs enrolled. Because IFN-γ production tends to decline as the disease progresses [6,16], evaluating sick dogs is crucial for effective disease monitoring. Therefore, other studies should further investigate the potential impact of the underrepresented sick group (groups 3 and 4) on the performance of WBA-T.

Furthermore, the use of WBA-T not only allows us to obtain IFN-γ results from dogs with shorter incubation times, but also offers the advantage of a simple technique that requires minimal care. This makes the method suitable for use in basic laboratory settings, potentially allowing for a wider uptake of cytokine measurement in dogs. As a result, this approach could significantly improve the monitoring of T-cell responses in *L. infantum*-infected dogs, contributing to a better understanding of the infection and supporting the development of new treatments or vaccines.

## 5. Conclusions

In conclusion, the WBA-T 48 h incubation period emerged as the optimal duration within the WBA-T condition after LSA stimulation.

Consequently, a 48 h WBA-T protocol can be deemed appropriate for assessing IFN-γ production in a clinical setting due to its favorable attributes, including efficiency, cost-effectiveness, and straightforward implementation.

## Figures and Tables

**Table 1:** Results of signalment and immunological tests performed in each group of dogs studied.

States of *L. infantum* Infection	Number of Dogs	Number of Dogs by Sex	Age (Years) (Median (Q1–Q3))	LSA IFN-γ (pg/mL) (WBA-S) (Median (Q1–Q3))	Serology (EU) (Median (Q1–Q3))
Healthy seronegative non-IFN-γ producers	14	♀: 5 ♂: 9	4 (3–7)	0 (0–13.95)	4.21 (2.55–6.24)
Healthy seronegative/seropositive IFN-γ producers	20	♀: 9 ♂: 11	5.5 (4–7)	821.65 (392.98–3117.5)	15.86 (11.81–32.4)
Sick non-IFN-γ producers	2	♀: 0 ♂: 2	9 (9–9)	0 (0–0)	279.81 (275.35–284.26)
Sick IFN-γ producers	5	♀: 2 ♂: 3	5 (4–6)	458.4 (297.5–1632)	286.71 (205.69–357.92)
Total dogs	41	♀: 16 ♂: 25	4.5 (3–7)	297.5 (0–1067)	13.5 (4.66–55.9)

ELISA: Enzyme-Linked Immunosorbent Assay, EU: ELISA units, IFN- γ: Interferon Gamma, LSA: *L. infantum* soluble antigen, Q1–Q3: First and third quartile, WBA-S: Standardized Whole Blood Assay and WBA-T: Whole Blood Assay in tube.

**Table 2:** IFN-γ concentration (pg/mL) in WBA-T and WBA-S after LSA stimulation for each incubation time in each clinical group of dogs.

IFN-γ pg/mL (Median (Q1–Q3)) After Stimulation with LSA					
States of *L. infantum* Infection (Health Status)	Infection Status	WBA-T 24 h (Median (Q1–Q3))	WBA-T 48 h (Median (Q1–Q3))	WBA-T 72 h (Median (Q1–Q3))	WBA-S 5 Days (Median (Q1–Q3))
Healthy seronegative non-IFN-γ producers	Non-infected	0 (0–9.79)	0 (0–37.46)	0 (0–57.45)	0 (0–13.95)
Healthy seronegative/seropositive IFN-γ producers	Infected	817.8 (82.3–2051.25)	1520.67 (234.03–2730.25)	1011.75 (295.05–1685.75)	821.65 (392.98–3117.5)
Sick non-IFN-γ producers	Infected	0 (0–0)	95 (47.5–142.5)	169.6 (84.8–254.4)	0 (0–0)
Sick IFN-γ producers	Infected	675.7 (631.7–3307)	976.9 (786–2692)	644.1 (624.8–1583.96)	458.4 (297.5–1632)

IFN- γ: Interferon Gamma, LSA: *Leishmania infantum* soluble antigen, Q1-Q3: First and third quartile, WBA-S: Standardized Whole Blood Assay and WBA-T: Whole Blood Assay in tube.

**Table 3:** IFN-γ concentration (pg/mL) in WBA-T and WBA-S after ConA stimulation for each incubation time in each clinical group of dogs.

IFN-γ pg/mL (Median (Q1–Q3)) After Stimulation with ConA					
States of *L. infantum* Infection (Health Status)	Infection Status	WBA-T 24 h (Median (Q1–Q3))	WBA-T 48 h (Median (Q1–Q3))	WBA-T 72 h (Median (Q1–Q3))	WBA-S 5 Days (Median (Q1–Q3))
Healthy seronegative non-IFN-γ producers	Non-infected	1367.5 (699.03–9287.25)	1298 (1087.58–7106)	2040.35 (796.4–7916.75)	8071.5 (5408–15,034.74)
Healthy seronegative/seropositive IFN-γ producers	Infected	8271.84 (3975.2–10,914.42)	7813.25 (4839.75–10,346.47)	8074.8 (4307.25–13,696.88)	14,260 (8392.83–19,317.5)
Sick non-IFN-γ producers	Infected	1791.4 (1093.6–2489.2)	2959.85 (1646.28–4273.43)	3230.1 (1784.65–4675.55)	1321.55 (970.33–1672.78)
Sick IFN-γ producers	Infected	3739 (2733–5361)	4176 (3409–7396.9)	4924 (3912.96–7627)	9916.06 (9393–11,090)

ConA: Concanavalin-A, IFN-γ: Interferon Gamma, Q1–Q3: First and third quartile, WBA-S: Standardized Whole Blood Assay and WBA-T: Whole Blood Assay in tube.

**Table 4:** Frequencies and results of IFN-γ production after stimulation with LSA at different incubation times from IFN-γ producer dogs.

Parameters (Units)	WBA-T 24 h	WBA-T 48 h	WBA-T 72 h	WBA-S 5 Days
Number of IFN-γ producer dogs (*n* = 25) *	20/25	22/25	20/25	25/25
Percentage (%)	80%	88%	80%	100%
Median (Q1–Q3) (pg/mL)	756.5 (109.8–2178)	1454 (319.9–2762)	1006 (292.3–1712)	820.8 (362.9–2482)

* IFN-γ producer dogs include healthy seronegative or seropositive dogs (group 2, *n* = 20) and sick seropositive dogs (group 4, *n* = 5). IFN-γ: Interferon Gamma, LSA: *Leishmania infantum* soluble antigen, Q1–Q3: First and third quartile, WBA-S: Standardized Whole Blood Assay and WBA-T: Whole Blood Assay in tube.

**Table 5:** Frequencies and results of IFN-γ production after stimulation with ConA (positive control) in different incubation times from all dogs studied.

Parameters (Units)	WBA-T 24 h	WBA-T 48 h	WBA-T 72 h	WBA-S 5 Days
Number of IFN-γ producer dogs (*n* = 41)	40/41	41/41	41/41	41/41
Percentage (%)	97.5%	100%	100%	100%
Median (Q1–Q3) (pg/mL)	4050 (1368–10,306)	5587 (1298–9985)	4924 (2118–10,858)	10,970 (6216–17,499)

ConA: Concanavalin-A, IFN-γ: Interferon Gamma, Q1–Q3: First and third quartile, WBA-S: Standardized Whole Blood Assay and WBA-T: Whole Blood Assay in tube.

## Data Availability

The original contributions presented in the study are included in the article/Supplementary Material, further inquiries can be directed to the corresponding authors.

## Supplementary Materials

The following supporting information can be downloaded at: https://www.mdpi.com/article/10.3390/ani14233464/s1, Table S1: signalment, clinical data and IFN-γ concentrations with WBA-T at 24, 48 and 72 h of incubation and WBA-S at 5 days of incubation after stimulation with LSA and ConA.
